# Role of the soluble epoxide hydrolase in the hair follicle stem cell homeostasis and hair growth

**DOI:** 10.1007/s00424-022-02709-4

**Published:** 2022-06-01

**Authors:** Zumer Naeem, Sven Zukunft, Stephan Günther, Stefan Liebner, Andreas Weigert, Bruce D. Hammock, Timo Frömel, Ingrid Fleming

**Affiliations:** 1 Institute for Vascular Signalling, Centre for Molecular Medicine, Goethe University, Theodor-Stern-Kai 7, 60590 Frankfurt am Main, Germany; 2 Bioinformatics and Deep Sequencing Platform, Max Planck Institute for Heart and Lung Research, 61231 Bad Nauheim, Germany; 3 Institute of Neurology (Edinger-Institute), Goethe-University Frankfurt, 60528 Frankfurt am Main, Germany; 4 Institute of Biochemistry I, Goethe-University Frankfurt, 60590 Frankfurt am Main, Germany; 5 Department of Entomology and Nematology and Comprehensive Cancer Center, University of California, Davis, CA USA; 6 German Center of Cardiovascular Research (DZHK), Partner site RheinMain, Frankfurt am Main, Germany

## Abstract

Polyunsaturated fatty acids (PUFAs) are used as traditional remedies to treat hair loss, but the mechanisms underlying their beneficial effects are not well understood. Here, we explored the role of PUFA metabolites generated by the cytochrome P450/soluble epoxide hydrolase (sEH) pathway in the regulation of the hair follicle cycle. Histological analysis of the skin from wild-type and sEH^−/−^ mice revealed that sEH deletion delayed telogen to anagen transition, and the associated activation of hair follicle stem cells. Interestingly, EdU labeling during the late anagen stage revealed that hair matrix cells from sEH^−/−^ mice proliferated at a greater rate which translated into increased hair growth. Similar effects were observed in in vitro studies using hair follicle explants, where a sEH inhibitor was also able to augment whisker growth in follicles from wild-type mice. sEH activity in the dorsal skin was not constant but altered with the cell cycle, having the most prominent effects on levels of the linoleic acid derivatives 12,13-epoxyoctadecenoic acid (12,13-EpOME), and 12,13-dihydroxyoctadecenoic acid (12,13-DiHOME). Fitting with this, the sEH substrate 12,13-EpOME significantly increased hair shaft growth in isolated anagen stage hair follicles, while its diol; 12,13-DiHOME, had no effect. RNA sequencing of isolated hair matrix cells implicated altered Wnt signaling in the changes associated with sEH deletion. Taken together, our data indicate that the activity of the sEH in hair follicle changes during the hair follicle cycle and impacts on two stem cell populations, i.e., hair follicle stem cells and matrix cells to affect telogen to anagen transition and hair growth.

**Supplementary information:**

The online version contains supplementary material available at 10.1007/s00424-022-02709-4.

## Introduction

Cytochrome P450 (CYP) enzymes are probably best known for their role in xenobiotic metabolism and steroid hormone biosynthesis, but they also metabolize endogenous polyunsaturated fatty acids (PUFAs) to generate intracellular mediators with biological activity [1–3]. Perhaps the most studied such metabolites are the epoxides of arachidonic acid: the epoxyeicosatrienoic acids, generated by CYP2C and CYP2J in several tissues including the brain, kidney vasculature, heart, and skin. Levels of the CYP-derived PUFA epoxides are tightly regulated by the activity of epoxide hydrolases, which convert the epoxides to their vicinal diols [4]. There are four members of the epoxide hydrolase family, but it is the soluble epoxide hydrolase (sEH) that seems to be the most important regulator of epoxide levels in vivo [5].

Numerous CYP enzymes have been detected in various types of stem and progenitor cells [6–8]. One good example being Cyp26b1 which is required to degrade retinoic acid in the skin and to promote hair follicle morphogenesis [9]. However, most studies only documented CYP expression or activity without determining the actions of specific PUFA metabolites [10, 11]. The sEH is also expressed in stem and progenitor cells in zebrafish embryos and its downregulation impaired the development of the caudal vein plexus [12], which serves as a transient hematopoietic tissue [13]. The lack of sEH activity decreased the numbers of lmo2/cmyb-positive progenitor cells, an effect that was rescued by the application of the sEH products; 12,13-dihydroxyoctadecenoic acid (12,13-DiHOME) and 11,12-dihydroxyicosatrienoic acid, apparently by stimulating canonical Wnt signaling [12]. In mice, sEH was detected in the bone marrow and its inactivation attenuated progenitor cell proliferation and mobilization as well as spleen colony formation. These effects contributed to the impaired functional recovery of sEH^−/−^ mice following hindlimb ischemia, which was rescued following either the restoration of bone marrow sEH activity or treatment with 12,13-DiHOME [12]. Thus, it seems that sEH activity is required for optimal progenitor cell proliferation, whereas long-term sEH inhibition maybe detrimental to progenitor cell proliferation, mobilization, and vascular repair.

One organ with a high stem cell proliferation and renewal capacity is the skin, and in particular hair follicles [14]. CYP enzymes and epoxide hydrolases are expressed in the skin [15–19], but little is known about the role of PUFA-derived mediators generated by CYP and sEH on hair follicle stem cell (HFSCs) or hair matrix cell proliferation to regulate hair growth. There is indirect evidence for such a role, as a polyphenol extract used to prevent chemotherapy-induced alopecia, increased the generation of the sEH products [20]. The aim of this study was, therefore, to investigate role of the sEH and its metabolites in the regulation of the hair follicle cycle and hair growth. Both the sEH (*Ephx2*) and epoxide hydrolase 3 (*Ephx3*) are reported to be present in the human and murine skin [15, 17, 19]. However, we focused only on the sEH given that Ephx3^−/−^ mice demonstrate no changes in the skin epoxide to diol ratio profile versus wild-type mice [21].

## Materials and methods

### Materials

The sEH inhibitors 4-[[trans-4-[[(tricyclo[3.3.1.13,7]dec-1-ylamino)carbonyl]amino]cyclohexyl]oxy]-benzoic acid (*t-*AUCB) and N-[1-(1-oxopropyl)-4-piperidinyl]-N'-[4-(trifluoromethoxy)phenyl]urea (TPPU) were synthesised as described [13, 22].

### Animals

C57BL/6(N) mice were purchased from Charles River (Sulzfeld, Germany) and TOPGAL/C57Bl6 transgenic mice harboring a β-galactosidase gene under the control of a LEF/TCF and β-catenin inducible promoter [23] were from the Jackson Laboratory (Bar Harbor, Maine). Floxed sEH mice (Ephx2tm1.1Arte on the C57BL/6(N) background) were generated by TaconicArtemis GmbH (Cologne, Germany) as described [24] and crossed with animals expressing Cre under the control of the endogenous Gt(ROSA)26Sor promoter (TaconicArtemis GmbH) to generate mice lacking the sEH in all tissues (sEH^−/−^). All animals were housed in conditions that conform to the guide for the care and use of laboratory animals in accordance with EU Directive 2010/63/EU as amended by Regulation (EU) 2019/1010 for animal experiments. Both the University Animal Care Committee and the Federal Authority for Animal Research at the Regierungspräsidium Darmstadt (Hessen, Germany) approved the study protocol (#FU 2001). For the isolation of organs, mice were sacrificed using 4% isoflurane in air followed by cervical dislocation. All of the pups studied were bred in the animal facility at the Goethe University Frankfurt, but the wild-type and sEH^−/−^ mice were not always littermates. While all the sEH^−/−^ were bred in house, adult wild-type mice were from Charles River. Male and female mice were used throughout.

To assess follicle cell proliferation, mice were given EdU (50 µg/g, i.p.; # 900,584, Sigma-Aldrich, Darmstadt, Germany) 6 h before sacrifice and harvesting the skin. In some experiments assessing hair growth, dorsal or whisker hairs were trimmed and the skin was treated topically with vehicle (25 µL DMSO in ∼250 μL Transderma Plo Gel Ultramax Base #TR220, Transderma Pharmaceuticals, Coquitlam, Canada) or the sEH inhibitor (TPPU; 30 µmol/L in 250 µL) in Transderma Plo Gel Ultramax Base as indicated in the “Results” section.

### Morphogenesis of hair follicle and progression of hair follicle cycle from resting to growth to destruction phase wild type vs sEH^−/−^

Skin samples were collected on postnatal days 3 to 32 of the physiological first and second hair cycles. The hair cycle in adult mice (7–8 weeks old) was synchronized and induced by depilation the dorsal skin. Animals were treated with vehicle (0.3% ethanol) or sEH inhibitor (*t*-AUCB, 8 mg/L) in the drinking water. Animals were photographed every other day after depilation, and skin pigmentation was monitored over 3 weeks to follow telogen to anagen progression, as described [25]. Thereafter, dorsal skin samples were harvested and either snap-frozen in liquid nitrogen or fixed for histological analyses. Hair follicle development was determined as described [26].

### Histological analysis and immunohistochemistry

Murine skin samples were fixed with 4% paraformaldehyde overnight at 4 °C. After fixation samples were dehydrated with an ethanol to xylol serial wash and embedded in paraffin. Sections (5 µm) were cut, deparaffinized, and rehydrated. For EdU incorporation, staining was performed using the Click-iT EdU Alexa Fluor 488 staining Kit (#C10337, ThermoFischer, Dreieich, Germany) according to the manufacturer’s instructions. Imaging was performed using a confocal microscope (LSM 780, Zeiss, Kelsterbach, Germany). Figures were prepared and analyzed using Imaris software (version 9.6.0 Bitplane AG, Zurich, Switzerland).

For histological analysis paraffin Sects. (5 µm) were dewaxed and then rehydrated in graded series of ethanol. Afterwards sections were stained with hematoxylin (Gill No. 3, GHS316, Sigma-Aldrich, Darmstadt, Germany) for 3 min, washed in running water for 10 min followed by 30 s staining of eosin (#R03040, Sigma-Aldrich, Darmstadt, Germany). Samples were then dehydrated and mounted with entallon (# 1.07960, Sigma-Aldrich, Darmstadt, Germany). Imaging was performed using an Axio Scope microscope (Zeiss, Kelsterbach, Germany). Figures were prepared using ImageJ (1.552a; Wayne Rasband, NIH, USA).

### Measurement of skin pigmentation area, hair follicle, and hair shaft length

Hair follicle length was measured using digital images of hematoxylin and eosin (H&E)-stained sections and ImageJ. To measure the hair shaft length, hairs from dorsal side of the mice were plucked and imaged using SteReo Lumar.V12 (Zeiss, Kelsterbach, Germany). Later, hair length was measured from digital images with ImageJ software. To determine pigmentation, the pigmented area was calculated from mouse images using ImageJ software (ImageJ 1.52a [27]) and normalized to the total depilated area.

### Vibrissa culture

Whisker pads were removed from 7-week-old mice as described [25]. Fat covering the follicles was removed under a dissection microscope, before individual whiskers were plucked from the whisker pad. Immediately after isolation, the hair shaft was cut back to the follicle surface and transferred to a 48-well plate. Follicles were allowed to adhere for 1–2 min before culture medium was added. Samples were maintained in William’E medium (#22,551–089 Thermo Fisher Scientific GmbH, Dreieich, Germany) supplemented with hydrocortisone (10 ng/mL), insulin (10 µg/mL), 400 U/mL penicillin, 400 μg/mL, streptomycin, and glutamine (2 mmol/L). In some cases, samples were also treated with solvent (0.1% DMSO), FCS (5%), 12,13-EpOME (10 µmol/L, #52,450, Cayman Chemical, Tallinn, Estonia), 12,13-DiHOME (10 µmol/L, #10,009,832, Cayman Chemical, Tallinn, Estonia), or glycogen synthase kinase-3 inhibitor (10 µmol/L; # CHIR99021, Sigma-Aldrich, Darmstadt, Germany). Whisker follicles were imaged on days 0 and 3 and hair regrowth was analyzed from digital images using ImageJ software.

### sEH activity assay

Soluble epoxide hydrolase activity was determined using cytosolic cell lysates generated as previously described [12]. Briefly, reactions were performed using 5 μg protein at 37 °C for 10 min in 100 μL of potassium phosphate buffer (100 mmol/L, pH 7.2) and started by the addition of 12,13-EpOME (0.25 μmol/L). Reactions were stopped by placing on ice, and samples were immediately extracted twice with ethyl acetate (0.5 mL). For liquid chromatography–tandem mass spectrometry (LC–MS/MS), one-tenth of the sample was spiked with a deuterated internal standard ((d4) 12,13-DiHOME). After evaporation of the solvent in a vacuum block under a gentle stream of nitrogen, the residues were reconstituted in 50 μL of acetonitrile to water (1:1, v/v) and determined with a Sciex API4000 mass spectrometer operating in multiple reaction monitoring (MRM) mode. Chromatographic separation was performed on a Gemini C18 column (150 × 2 mm I.D., 5-μm particle size; Phenomenex, Aschaffenburg, Germany).

### Measurement of PUFA metabolites

Skin samples (30–40 mg) or 20–25 isolated hair follicles were ground under liquid nitrogen and lysed in homogenization buffer (50 mmol/L Tris–HCL, 0.25 mol/L sucrose, 2 mmol/L EDTA, 150 mmol/L KCl together with fresh 0.25 mmol/L phenylmethylsulfonyl fluoride, and 1 mmol/L dithiothreitol. Samples were spiked with a deuterated internal standard mix (8,9-DHET-d11, 11,12-DHET-d11, 14,15-DHET-d11, 9,10-DiHOME-d4, 12,13-DiHOME-d4, 5,6-EET-d11, 8,9-EET-d8, 11,12-EET-d8, 14,15-EET-d8, 9.10-EpOME-d4, 12,13-EpOME-d4, 5S-HETE-d8, 12S-HETE-d8, 15S-HETE-d8, 20-HETE-d6, 9S-HODE-d4, 13S-HODE-d4; Cayman Chemical, Tallinn, Estonia). Samples were extracted with 1 mL ethyl acetate, vortexed for 20 s, placed on ice for 10 min, vortexed again, and subsequently centrifuged (10,000 g, 5 min, 4 °C). The upper phase was collected, and the ethyl acetate extraction was performed again in 0.5 mL. Both upper phase samples were combined and evaporated to dryness in a vacuum manifold under a continuous nitrogen stream. The residues were reconstituted with 50 μL of acetonitrile to water (50:50, v/v, containing 100 ng/mL flufenamic acid as internal control) and determined with a Sciex QTrap5500 mass spectrometer operating in MRM (negative ionization) mode. ESI parameters were set to CUR: 24 psi, IS: -4500 V, TEM: 600 °C, GS1: 45 psi, GS2: 60 psi. Chromatographic separation was performed on an Agilent 1290 Infinity LC system (Agilent, Waldbronn, Germany), using a Gemini C18 column (150 × 2 mm I.D., 5 μm particle size; Phenomenex, Aschaffenburg, Germany). The mobile phase consisted of (A) water + 0.0125% ammonia and (B) acetonitrile + 0.0125% ammonia. Elution of analytes was carried out under gradient conditions at a flow rate of 0.5 mL/minute going from 15% B to 40% B in 10 min, increasing to 90% B in 2 min, hold 90% B for 1 min, and equilibrated in 15% B for 5.5 min. Ten microliters of each sample was injected onto the column. The column temperature was kept at 40 °C. Samples were kept in the auto-sampler at 6 °C until analysis. For the preparation of calibration curves, stock solutions were prepared in ethanol that contained primary fatty acids and oxylipin standards. Working standard solutions were prepared by serial dilution of the stock solutions to create the necessary concentrations. All samples and dilutions of the standards were spiked with internal deuterated standards: Metabolite concentrations were determined by reference to the standards. Analyst 1.6.2 and MultiQuant 3.0 (both Sciex, Darmstadt, Germany) were used for data acquisition and analysis, respectively. Analyzed data was normalized to the weight of the skin.

### Skin digestion, hair matrix cell sorting

To generate a single-cell suspension from the skin, subcutaneous fat was removed, and the skin was chopped into 1-mm pieces which were then incubated in liberase (300 µg/mL; Cat# 5,401,119,001, Sigma-Aldrich, Darmstadt, Germany) and DNase 1 digestion cocktail (50 U/mL, Cat# Roche-11284932001, Sigma-Aldrich, Darmstadt, Germany) for 60–90 min at 37 °C as described [28]. Digested skin cells were passed through a 100-µm pore filter and then through a 40-µm pore filter. The single cells obtained were washed once in 1% BSA and then blocked with FcR blocking reagent (Cat# 101,302, BioLegend, San Diego, USA) for 10 min. Thereafter, cells were incubated with following antibodies; anti-P-cadherin (Cat# AF761, R&D Systems, Minnesota, USA), anti-CD49f-APC (Cat# 17–0495-82, Invitrogen, Frankfurt, Germany), anti-CD45-Vio-Blue (Cat# 130–092-910, Miltenyi Biotec, Bergisch Gladbach, Germany), anti-CD-90.2-PE (Cat# 130–102-489, Miltenyi, Bergisch Gladbach, Germany), and CD31 (Cat#DIA 310, clone: SZ31, Dianova, Hamburg, Germany). An Aria III cell sorter (BD Biosciences, Heidelberg, Germany) was used to sort the hair matrix cells (PCAD + CD49f + CD45-CD90.2- CD31-CD326-).

### RNA sequencing

RNA was extracted from the sorted matrix cells with TRIzol LS reagent (Cat# 10,296,010, ThermoFisher Scientific, Frankfurt, Germany). In column DNase, digestion was performed to eliminate DNA contamination. RNA and library preparation integrity were verified with LabChip Gx Touch 24 (Perkin Elmer). Total RNA (8 ng) was used as input for Pico Input Mammalian (Takara Biom). Sequencing was performed on the NextSeq2000 instrument (Illumina, Shirley, New York) using P3 flowcell with v3 chemistry, resulting in average of 35 M reads per library with 1 × 72 bp single end setup. The resulting raw reads were assessed for quality, adapter content, and duplication rates with FastQC (http://www.bioinformatics.babraham.ac.uk/projects/fastqc). Trimmomatic version 0.39 was employed to trim reads after a quality drop below a mean of Q20 in a window of 10 nucleotides [29]. Only reads between 30 and 75 nucleotides were cleared for further analyses. Trimmed and filtered reads were aligned versus the Ensembl mouse genome version mm10 (Ensembl release 101) using STAR 2.7.7a with the parameter “–outFilterMismatchNoverLmax 0.1” to increase the maximum ratio of mismatches to mapped length to 10% [30]. The number of reads aligning to genes was counted with featureCounts 1.6.5 tool from the Subread package [31]. Only reads mapping at least partially inside exons were admitted and aggregated per gene. Reads overlapping multiple genes or aligning to multiple regions were excluded. Differentially expressed genes were identified using DESeq2 version 1.30.0 [6]. Only genes with a minimum fold change of +—1.5 (log2 + -0.59), a maximum Student’s *t* test corrected *P* value of 0.05, and a minimum combined mean of 3 reads were deemed to be significantly differentially expressed. The Ensemble annotation was enriched with UniProt data (release 24.03.2017) based on Ensembl gene identifiers.

### Statistical analysis

Data are expressed as mean ± SEM. The statistical evaluation was performed using Student’s *t* test for unpaired data, one-way ANOVA followed by Tukey’s multiple comparisons test or ANOVA for repeated measures where appropriate. Values of *P* < 0.05 were considered statistically significant.

## Results

### Effect of sEH deletion on the hair follicle cycle progression

**Fig. 1:**
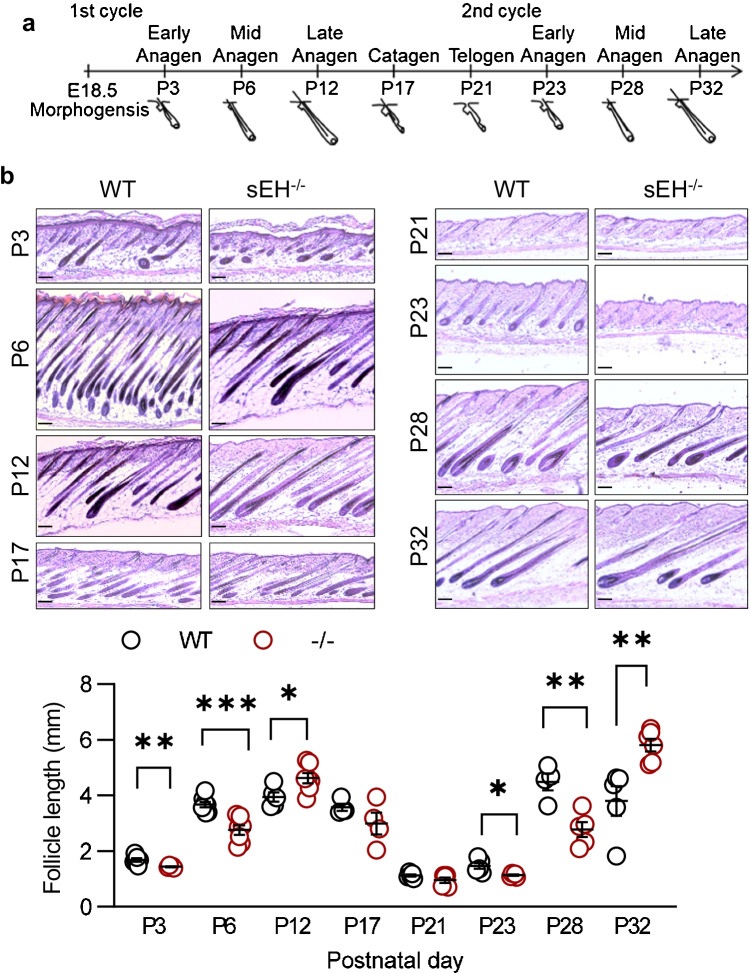
Effect of sEH deletion on hair follicle cycle progression. **a** Scheme adapted from reference [33] showing the hair follicle cycle. **b** H&E staining of the dorsal skin from wild-type (WT) and sEH^−/−^ (-/-) mice from P3 to P32; bar = 100 µm; n = 4–6 mice per time point; 15–20 hair follicles per mouse (Student’s t test). *P < 0.05, **P < 0.01, ***P < 0.001

The hair follicle cycles through sequential phases of active regeneration and hair growth (anagen), destruction (catagen), and then rest (telogen) [32]. To assess the potential role of the sEH in hair follicle cycle progression, the dorsal skin from wild-type and sEH^−/−^ mice was compared on postnatal days (P) 3 to P32 (Fig. 1a). Comparative histology revealed significant differences in hair follicle elongation between the two genotypes during the early to mid-anagen phases (P3 and P6), with follicle growth in sEH^−/−^ mice lagging behind that of wild-type mice. Similar results were obtained during the onset of the second growth phase (P23 and P28) (Fig. 1b). However, the situation was reversed during the late anagen stage so that the follicles in the skin from sEH^−/−^ mice were longer than the follicles in the skin from wild-type mice. Hair follicle length during the subsequent regression (P17) and quiescent periods (P21) was comparable in sEH^−/−^ and wild-type mice.

### sEH and the proliferation of hair follicle stem cells

**Fig. 2:**
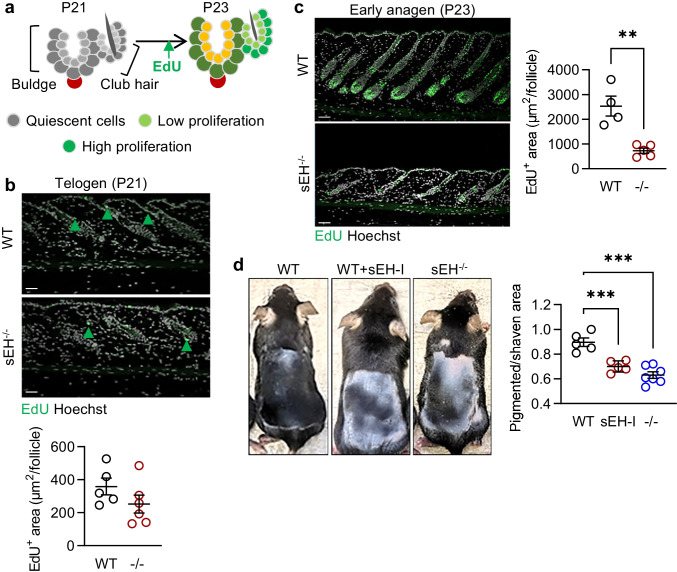
sEH deletion inhibits hair follicle stem cell activation and delays the telogen to anagen transition. **a** Scheme, adapted from [36], showing telogen (P21) and anagen (P23) transition. **b, c** EdU staining (green) in the dorsal skin of wild-type (WT) and sEH^−/−^ (-/-) mice during the telogen (**b**, green arrow heads indicate the weak EdU signal) and early anagen (**c**) stages, bar = 50 µm; *n* = 4–6 mice per group, 15–20 follicles per mouse (Student’s *t* test). **d** Skin pigmentation as an indicator of telogen to anagen transition in wild-type mice treated with vehicle or the sEH inhibitor (sEH-I) *t*-AUCB and sEH^−/−^ mice treated with vehicle only; *n* = 5–10 mice per group (one-way ANOVA and uncorrected Fisher’s LSD test). *P < 0.05, **P < 0.01, ***P < 0.001

During the telogen to anagen transition (P21-P23), HFSCs exit quiescence and start to proliferate (see Fig. 2a). To determine whether the sEH affects the latter process, HFSC proliferation was monitored by administering EdU 6 h before harvesting the skin. As expected, little or no proliferation was detected during the resting stage (P21) (Fig. 2b). On P23, however, there was a clear incorporation of EdU into HFSCs in the skin from wild-type mice that exceeded that detected in sEH^−/−^ mice (Fig. 2c). Further proliferation chasing experiments during the mid-anagen stages (P3 and P28) also revealed fewer EdU + cells in the skin from sEH^−/−^ mice (Supplementary Fig. 1). These observations indicate that the sEH is required for efficient HFSC activation and proliferation.

In C57BL/6 mice, melanocyte stem cells proliferate together with HFSCs during the anagen phase before differentiating into mature melanocytes [33]. Melanin produced by the latter cells is transported to surrounding keratinocytes resulting in the pigmentation of newly formed hairs and otherwise nude skin, which makes skin color a good indicator of hair follicle stages [32, 34, 35]. To investigate the effects of sEH deletion on HFSC activation and anagen progression, hair regrowth was assessed using a depilation-induced synchronization of the hair follicle cycle that involved depilation during the second telogen stage (P49) and analysis of hair regrowth during subsequent anagen stage (P68). This revealed that telogen to anagen transition, as accessed by skin pigmentation, was significantly delayed in sEH^−/−^ animals (Fig. 2d). Similarly, when wild-type mice were given a sEH inhibitor in the drinking water (*t-*AUCB, 8 mg/L), a delay in skin pigmentation was also detected.

### sEH, proliferation of hair matrix cells and hair growth

**Fig. 3:**
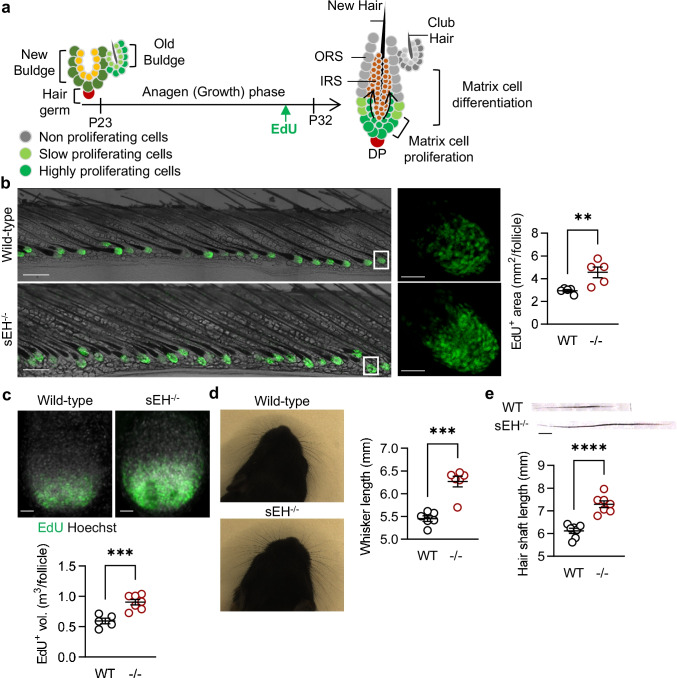
Consequences of sEH deletion on hair matrix cell proliferation and differentiation. **a** Scheme adapted from [36], showing the activation, proliferation, and differentiation of hair matrix cells during anagen stage progression. ORS, outer root sheet; IRS, inner root sheet; DP, dermal papillae. **b** Hair matrix cell proliferation (EdU + cells) in late anagen-staged skin (P32) from wild-type (WT) and sEH^−/−^ (-/-) mice, bar = 20 µm. The white boxes indicate the area magnified in the right hand panels, bar = 50 µm. *N* = 5 mice per group, with 10–15 follicles analyzed per mouse (Student’s *t* test). **c** EdU + cells in individual whiskers from wild-type (WT) and sEH^−/−^ (-/-) mice, bar = 50 µm; *n* = 5–8 mice per group; with 8 whisker follicles studied per mouse (Student’s *t* test). **d** Whisker length on P6 in wild-type (WT) and sEH^−/−^ (-/-) mice; *n* = 6 mice per group (Student’s *t* test). **e** Dorsal hairs on P12 from wild-type (WT) and sEH^−/−^ (-/-) mice, scale bar = 1 mm; *n* = 7 mice per group with 25–30 analyzed hairs per mouse (Student’s *t* test). **P < 0.01, ***P < 0.001

During the late growth stage, stem cells are quiescent and proliferation is restricted to the lower outer root sheet and hair matrix cells [36]. The latter are transit-amplifying cells derived from the HFSCs that make the inner root sheet and the new hair shaft [37]. Therefore, we analyzed the role of sEH on hair matrix cell proliferation by labeling with EdU during the late growth phase (Fig. 3a). This revealed that hair matrix cell proliferation was significantly greater in the skin from sEH^−/−^ than from wild-type mice (Fig. 3b). EdU incorporation into hair matrix cells in growing whiskers was also greater in sEH^−/−^ mice. (Fig. 3c). This translated into altered hair growth as the length of both the whiskers (Fig. 3d), and plucked dorsal hair (Fig. 3e) was greater in sEH^−/−^ mice. Overall, hairs were 15–20% longer in sEH^−/−^ versus wild-type mice.

**Fig. 4:**
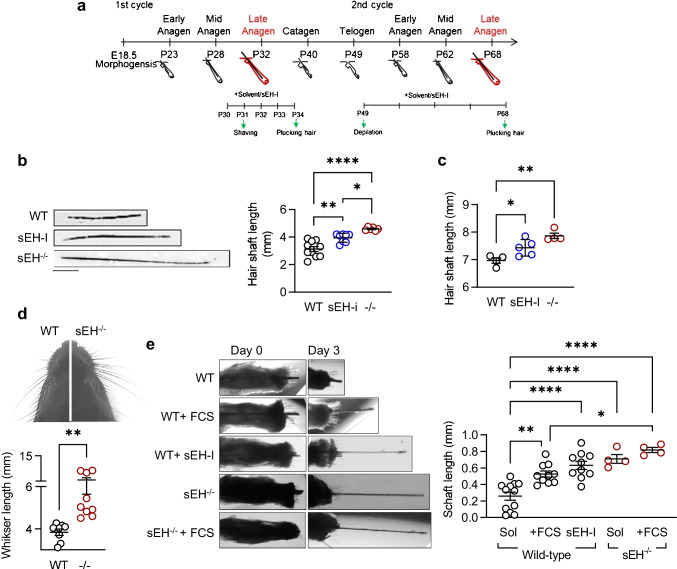
sEH inhibition and deletion accelerate hair growth. **a** Scheme showing the first and second hair follicle cycles as well as the harvesting and inhibitor treatment protocols. **b** Re-grown dorsal hair from wild-type (WT) mice treated with vehicle (25 µl DMSO in 250 µL PLO Base) or the sEH inhibitor (sEH-I) TPPU (dissolved in DMSO and then added to 250 µl PLO Base to give a final concentration of 30 µmol/L), and sEH^−/−^ (-/-) mice for 4 days after shaving (i.e., on P31); *n* = 6–10 mice per group with 15 hairs per animal, scale bar = 1 mm (one-way ANOVA and uncorrected Fisher’s LSD test). **c** Quantification of hairs from the dorsal skin of wild-type (WT) and sEH^−/−^ (-/-) mice treated with vehicle (0.3 Vol % ethanol in water) or a sEH-I (dissolved in ethanol and then added to 0.3% Vol in water to 8 mg/L final) for 4 weeks after depilation-induced synchronization of the second hair growth cycle; *n* = 4–5 mice per group with 15–20 hairs per mouse (one-way ANOVA and Tukey’s multiple comparison test). **d** Whisker regrowth in 7-week-old mice, 10 days after shaving; *n* = 8–9 mice per group (Student’s *t* test). **e** Whisker outgrowth from isolated vibrissae from wild-type and sEH^−/−^ mice cultured with solvent, 5% FCS or the sEH inhibitor (sEH-I; *t*-AUCB 10 µmol/L). Images were taken immediately after isolation (day 0) and after 3 days of culture; *n* = 4–12 mice per group with 3–4 whisker follicles per mouse (one-way ANOVA & Tukey’s multiple comparison test). *P < 0.05, **P < 0.01, ***P < 0.001, and ****P < 0.0001

To determine whether the contribution of the sEH to increased hair growth could be attributed to an effect on matrix cells versus a shift in the growth phase, hairs were synchronized by trimming during the late growth phase when proliferation is restricted to the lower root sheet and matrix cells [38, 39]. Mice were then treated topically with either solvent or the sEH inhibitor; TPPU (30 µmol/L), for 4 days (Fig. 4a). Over this short time, hair outgrowth was faster in animals that either lacked the sEH or were treated topically with the sEH inhibitor; TPPU (Fig. 4b). A similar effect was also observed when the dorsal skin was depilated on P49, i.e., during the telogen stage (Fig. 4c). Also, whisker regrowth in 7-week-old mice was significantly greater in sEH^−/−^ mice 10 days after trimming, with hairs approximately 2.6 fold longer than wild-type mice (Fig. 4d). To assess the impact of sEH deletion in the vibrissae (whisker) follicles and rule out an influence of other factors or infiltrating cells, follicles were isolated from whisker pads of wild-type and sEH^−/−^ mice and hair growth was monitored over 3 days in vitro. Hair growth from wild-type vibrissae was slow in the presence of solvent but significantly increased in the presence of FCS (Fig. 4e). The addition of *t*-ACUB further increased the length of the hair shaft to values comparable with those detected in vibrissae from sEH^−/−^ mice.

### Cycle-dependent changes in sEH activity and its respective effects on oxylipin profile of the skin

**Fig. 5:**
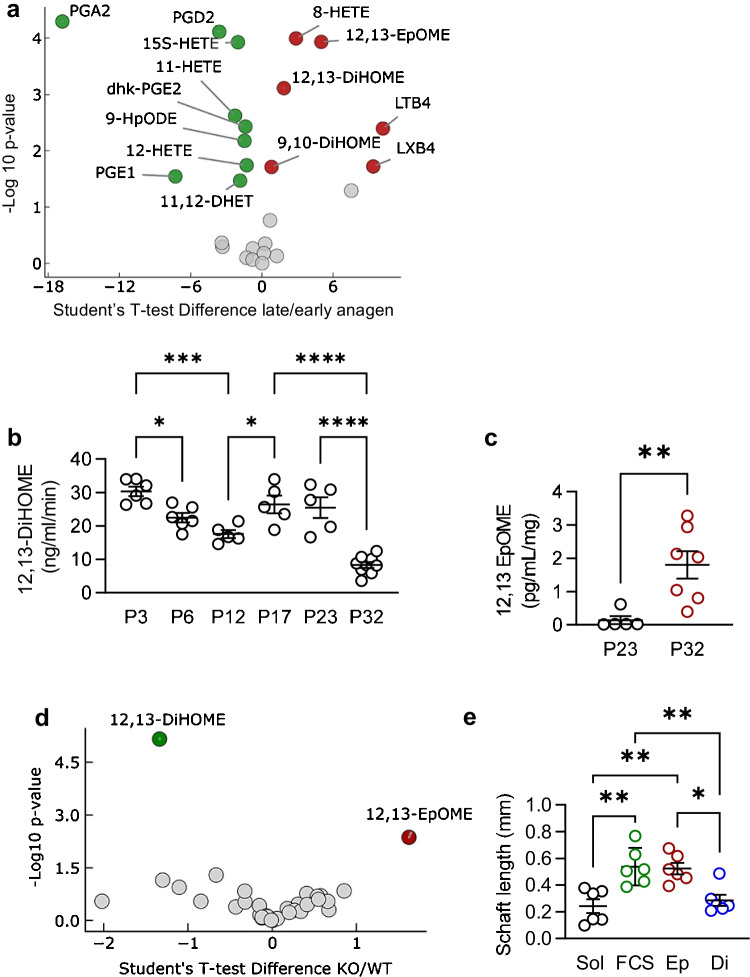
Effect of sEH deletion on the lipid profile of the dorsal skin. **a** Volcano plot comparing PUFA-derived mediators detected in the dorsal skin of wild-type (WT) mice from early (P23) vs late anagen phase (P32); *n* = 5–8 mice per group. Green = higher in P23, red = higher in P32, and gray = no significant difference between stages. **b** sEH activity (12,13-DiHOME generation) in the dorsal skin from wild-type mice from the early growth phase to the late growth phase of the first and second hair follicle cycles; *n* = 5–9 mice per group (one-way ANOVA and Tukey’s multiple comparisons test). **c** Levels of 12,13-EpOME from the same dataset as in **b**; *n* = 5–8 mice per group (Student’s *t* test). **d** Volcano plot comparing PUFA-derived metabolites in isolated murine whisker follicles from wild-type (WT) and sEH^−/−^ (-/-) mice; *n* = 4–5 mice per group and 25 follicles per mouse. **e** Comparison of the effects of solvent (Sol), 5% FCS, 12,13-EpOME (Ep), and 12,13-DiHOME (Di; both 10 µmol/L) on the in vitro growth of vibrissae from wild-type mice; *n* = 6 mice; 3–4 whisker follicles per mouse (one-way ANOVA and Tukey’s multiple comparison test). *P < 0.05, **P < 0.01, ***P < 0.001, and ****P < 0.0001

As the sEH metabolized PUFA epoxides to diols, the skin was harvested from wild-type mice during the early (P23) and late anagen (P32) phases and changes in PUFA-derived mediators were assessed by LC–MS/MS. This approach revealed that the oxylipin content of the murine skin differed during different stages of the hair follicle cycle (Supplementary Fig. 2, Fig. 5a). While cyclooxygenase-derived metabolites, including prostaglandins, were enriched during the early growth phase, lipoxygenase-, and CYP to sEH-derived oxylipins were more abundant during the late anagen phase. Among the most altered PUFA mediators were the linoleic acid epoxide 12,13-epoxyoctadecenoic acid (12,13-EpOME) and its diol 12,13-DiHOME.

To strengthen the link between the different stages of the follicle cycle and the sEH, enzymatic activity was assessed in the dorsal skin by monitoring the in vitro conversion of 12,13-EpOME to 12,13-DiHOME. This approach revealed that sEH activity was high at the beginning of the growth phase (P3 and P23) but decreased during follicle maturation (P12 and P32) (Fig. 5b). Correspondingly, 12,13-EpOME levels were low in the skin on P23, i.e., when sEH activity was high and the increase in 12,13-EpOME levels on P32 coincided with a decrease in sEH activity (Fig. 5c). To determine whether the same PUFA-derived mediators were affected by sEH deletion, anagen stage hair follicles were isolated from whisker pads. LC–MS/MS profiling revealed that 12,13-EpOME and -DiHOME were the PUFA-derived mediators most altered by deletion of the sEH (Fig. 5d, Supplementary Fig. 3). To determine whether or not these PUFA-derived mediators impacted on hair growth, vibrissae from wild-type mice were cultured in vitro with either 12,13-EpOME or 12,13-DiHOME for 3 days. While the addition of 12,13-EpOME significantly increased hair shaft growth, 12,13-DiHOME had no effect (Fig. 5e).

### Impact of the sEH on gene expression in hair matrix cells

**Fig. 6:**
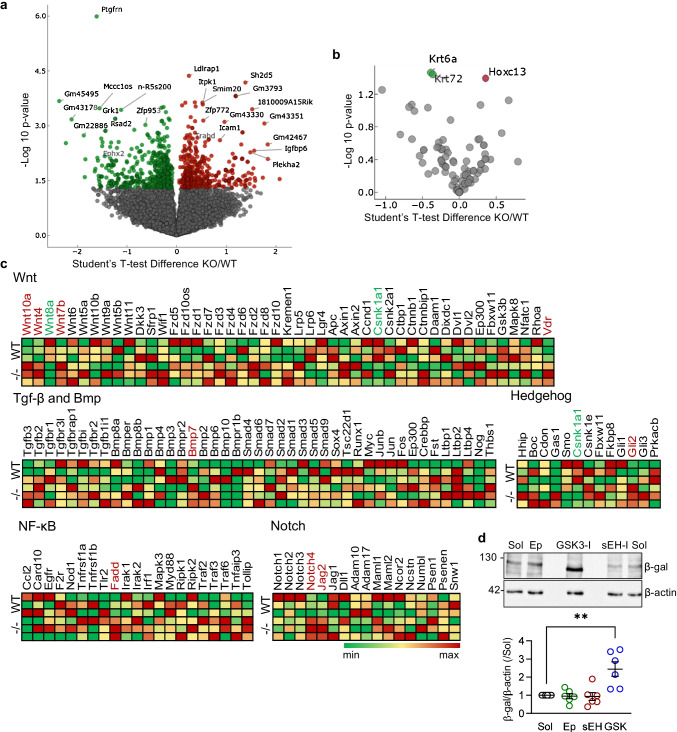
Consequence of sEH deletion on the genetic profile of hair matrix cells. **a** Volcano plot showing differentially expressed genes in sorted hair matrix cells from mature hair follicles (on P32) from wild-type (WT) and sEH^−/−^ (KO) mice; *n* = 3–4 per mice per group. **b** Volcano plot showing the matrix cell lineage-specific keratins and transcription factors altered in hair matrix cells from sEH^−/−^ (KO) versus wild-type (WT) mice; *n* = 3–4 per mice. **c** Heat maps comparing the expression of the genes in panel a linked to selected signaling pathways; *n* = 3 mice per group. **d** Comparison of β-galactosidase (β-Gal) expression in hair follicles from TOPGAL transgenic mice following treatment with solvent (Sol), 12,13-EpOME (Ep, 10 µmol/L), sEH-inhibitor (sEH, 10 µmol/L), or a GSK3-inhibitor (GSK, 10 µmol/L); *n* = 6 mice per group with each data point the mean of 7 follicles. **P < 0.01

To gain insight into the molecular events linking PUFA-derived mediators with molecular changes, transit-amplifying matrix cells were isolated (FACS sorting) from skin on P32, i.e., during the late anagen phase. RNA-sequencing identified a number of genes that were differentially regulated in skin from wild-type versus sEH-deficient mice. Among the downregulated transcripts were the sEH (*Ephx2*) and the prostaglandin (PG) F2 receptor inhibitor (*Ptgfrn*) (Fig. 6a, Dataset 1). While the decrease in *Ptgfrn* could not be confirmed at the protein level due to the lack of a specific antibody, it was coincident with an increase in PGF2α levels (Supplementary Fig. 4), a combination that would promote PGF2α signaling. Additional significantly altered transcripts were matrix cell lineage-specific keratins and transcription factors (Fig. 6b). Also, components of the Wnt signaling pathway, e.g., Wnt10a, Wnt4, Wnt7b, and Vdr, were consistently upregulated in sEH-deficient matrix cells while Wnt8b and Csnk1a1 were downregulated. Other pathways implicated in matrix cell proliferation were also consistently increased in sEH-deficient cells including Bmp7, Cli2, Fadd, Notch4, and Jag2 (Fig. 6c). As the most pronounced changes detected were in genes related to the Wnt pathway, we determined the effect of 12,13-EpOME on hair follicles isolated from the whisker pads of TOPGAL β-catenin reporter mice [23]. Treating hair follicles for 2 days with either solvent, 12,13-EpOME, or the sEH inhibitor *t-*AUCB had no effect on β-galactosidase expression while a glycogen synthase kinase-3 (GSK3)-inhibitor clearly increased the signal (Fig. 6d). Thus, the sEH-inhibitor- and 12,13-EpOME-induced increase in hair growth could not be clearly linked with β-catenin signaling.

## Discussion

The results of our investigation revealed that the activity of the sEH in hair follicle changes during the hair follicle cycle and impacts on two-stem cell populations, i.e., HFSCs and matrix cells, to affect telogen to anagen transition and hair growth. Deletion of the sEH has clear effects on the oxylipin content of the skin and isolated vibrissae and marked changes in levels of the linoleic acid epoxide; 12,13-EpOME. Indeed, 12,13-EpOME levels were increased in the mature versus the early growth phase hair follicles, when sEH activity was low. Moreover, the application of 12,13-EpOME, but not its diol and sEH product; 12,13-DiHOME, increased hair growth in vibrissae in vitro. These observations indicate that the sEH expression regulates local levels of 12,13-EpOME to regulate HFSC and matrix cell proliferation in hair follicles.

Low levels of linoleic acid are essential for maintaining skin barrier function [22], and their sEH-dependent metabolism to epoxides and diols as well as to linoleate triols in the epidermis implies a role in formation of the mammalian water permeability barrier [19]. PUFA-derived eicosanoids have not been explored extensively with respect to their effects on hair follicle stem cell and hair matrix cell proliferation to regulate hair growth. Nevertheless, animal and plant oils enriched with ω-3 and ω-6 PUFAs have potential use for the treatment of hair loss and hair growth [40–42]. Although mechanisms have not been studied in great detail, arachidonic acid and linoleic acid have been reported to increase the expression of a series of growth factors that promote hair growth [41, 42].

In mice, the first few hair cycles are synchronized which makes them a good model system to assess how different genes or factors affect the transition of stem cells through sequential cycles of quiescence and tissue regeneration [14]. Monitoring two follicle cycles revealed that the activity of the sEH was not constant and changed quite markedly during cycle progression. This cyclic change in activity was accompanied by a dual effect of sEH deletion on follicle cycle progression, i.e., an initial delay in entry into the follicle cycle followed by a recovery in follicle length that exceeded that seen in wild-type mice. Hair cycles are fuelled by the HFSCs in the ‘‘bulge’’ niche located at the base of the telogen phase hair follicle [43], and it was possible to detect sEH-dependent alterations in HFSC proliferation. Differences were most pronounced during the early anagen phase when HSFC proliferation in sEH^−/−^ mice lagged behind that in wild-type mice and was accompanied by delayed skin pigmentation. These findings suggest that sEH activity is linked to HFSC activation and telogen to anagen transition.

The mammalian sEH protein is a homodimer, and each monomer consists of an N-terminal domain which displays lipid phosphatase activity and a larger C-terminal which possesses classical α/β-hydrolase activity [44, 45]. Therefore, in a second approach to ensure that the defects observed in the sEH^−/−^ mice were due to the loss of epoxide hydrolase activity, wild-type mice were treated with an epoxide hydrolase inhibitor, i.e., *t*-AUCB [46]. A delay in skin pigmentation was also detected in wild-type mice treated with *t*-AUCB in the drinking water during the depilation-induced hair follicle cycle. Although we have not proposed a mechanism to account for our findings, it should be pointed out that the report linking sEH with stem cell proliferation in zebrafish and mice identified the activation of a Wnt signaling pathway [12]. This is of relevance as Wnt and BMP signals have a known role in the adult hair cycle [14].

In contrast to the attenuated proliferation of HFSCs, matrix cell proliferation was enhanced during the late anagen phase in sEH-deficient mice. These changes translated into increased growth of dorsal hair as well as whiskers (vibrissae) and could be attributed to local consequences of sEH deletion within the follicle. Indeed, isolated and cultured vibrissae also grew longer when either the sEH was not expressed or the enzyme was inhibited. Since sEH metabolizes PUFA epoxides to their vicinal diols [47], LC–MS/MS was used to determine which PUFA metabolites in whisker follicles were affected by sEH deletion. Clear changes in only one epoxide/diol pair, i.e., 12,13-EpOME/DiHOME were detected, and of these, only the sEH substrate; 12,13-EpOME, stimulated whisker growth. While some PUFA epoxides, in particular, the epoxides of arachidonic acid (the epoxyeicosatrienoic acids) are thought to act by binding to membrane bound receptors to activate protein kinase A [4], nothing is known about the molecular events that underlie the actions of 12,13-EpOME [48]. RNA sequencing of sorted matrix cells was performed to shed some light on the topic. This approach picked up a negative regulator of the prostaglandin F2 receptor, i.e., *Ptgfrn* as the most downregulated gene in sEH-deficient mice. As levels of PGF2α were also increased in skin from sEH^−/−^ mice, it seems that the lack of sEH can influence both levels of the agonist as well as the activity of the receptor to promote PGF2α signaling. This unexpected crosstalk between the sEH and prostaglandin levels is of interest given that the PGF_2α_ analogs latanoprost and bimatoprost show promise in the treatment of patterned hair loss and androgenic alopecia as well as hair re pigmentation [49, 50]. As interesting as the latter link is, it seems unlikely to account for all of the actions of 12,13-EpOME. The RNA-seq studies also picked up what seemed to be a link to Wnt signaling, that fit well with previous observation in zebrafish embryos and murine bone marrow [12], as well as previous observations relating to the role of Cyp26b1 in hair follicle development [9]. However, a clear sEH-dependent difference in the activation of β-catenin was not obvious, at least in isolated vibrissae in vitro, perhaps implicating the noncanonical rather than the canonical branch of that signaling pathway. It also remains to be determined whether transforming growth factor/bone morphogenic protein, Hedgehog, nuclear factor κB or Notch signaling pathways can be activated by 12,13-EpOME, as all have been implicated in the regulation of skin stem cell function [14, 51]. Matrix cells have received a lot of attention over the last few years as they are the main drivers of the anagen stage and hair growth [36, 52]. It even seems that these progenitor cells are mobilized in response to ionizing radiation and acquire a stem cell-like state to fuel hair follicle regeneration before homing back to their niche [53]. Given that sEH deletion and 12,13-EpOME treatment increased the proliferation of matrix cells, sEH inhibition could serve as a therapeutic option for treatment of hair loss at early stages of cancer therapy by prolonging the anagen stage.

Taken together, our studies have revealed a role for cyclic increases and decreases in sEH expression and activity in the coordination of the hair follicle cycle and HFSC proliferation that is closely linked to the levels of its substrate 12,13-EpOME, as well as in matrix cell proliferation and hair growth. Hair loss is more frequently suffered by males than females, and it is worth pointing out that sEH expression is higher in males than in females [54, 55]. While this can account for sex-associated differences in blood pressure, it remains to be determined whether or not a similar pattern can be detected in hair coverage. Indeed, although male and female mice were included in our study and we failed to observe any sex-dependent differences in the hair follicle cycle. However, we studied the follicle cycle in young mice and it will be interesting to determine whether changes in hair growth or loss become apparent between wild-type and sEH^−/−^ mice on aging.

## Supplementary information

Below is the link to the electronic supplementary material.

Supplementary file1 (PDF 437 KB)

## Data Availability

All data generated or analyzed during this study are included in this published article (and its supplementary information files).
