# Supplementary material for: Role of the soluble epoxide hydrolase in the hair follicle stem cell homeostasis and hair growth
Source: Pflugers Arch. 2022 Jun 1;474(9):1021–35. doi: 10.1007/s00424-022-02709-4 (PMC9393123; doi:10.1007/s00424-022-02709-4)
Supplement: Supplementary file 1 — Supplementary file1 (PDF 437 KB) [file 424_2022_2709_MOESM1_ESM.pdf]

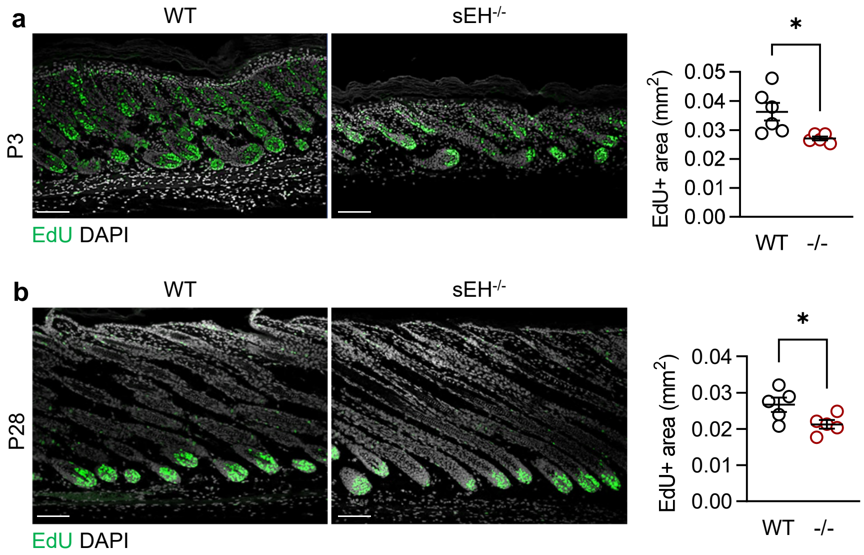

Supplementary Fig. 1. Consequences of sEH deletion on EdU incorporation into murine skin during mid anagen. EdU staining (green) in the dorsal skin of wild-type (WT) and $sEH^{-/-}$ (-/-) mice on (**a**) P3 and (**b**) P28, bar = 100 µm; n = 5-6 mice per group, with 15-20 follicles assessed per mouse (Student's t test). *P<0.05.

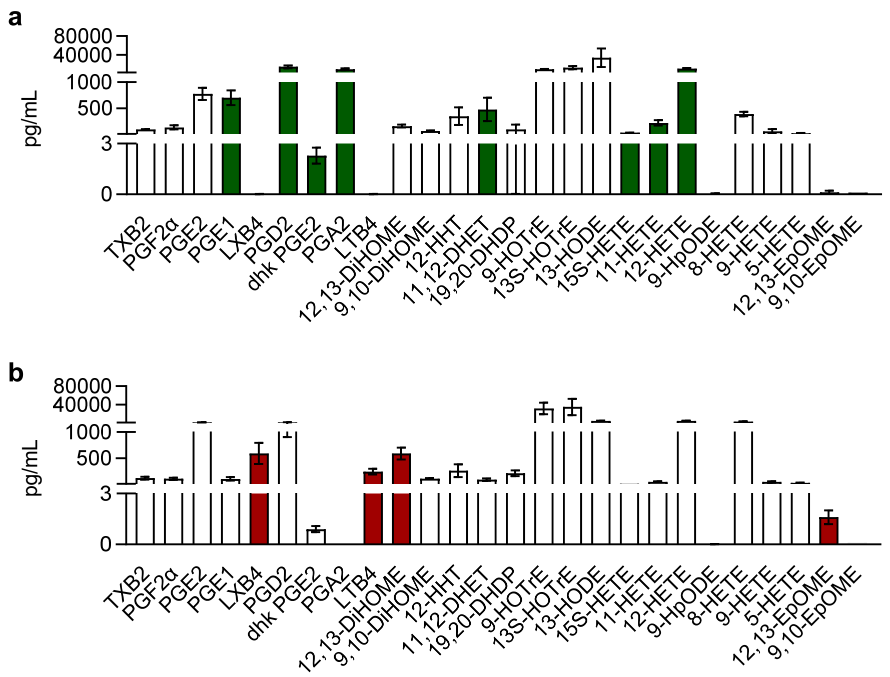

Supplementary Fig. 2. Comparison of the PUFA profile of skin in the early and late anagen stages. PUFA-derived mediators detected in the dorsal skin of wild-type mice from (**a**) the early (P23) and (**b**) the late anagen phase (P32); n=5-8 mice per group; Green bars indicate metabolites that were significantly higher on P23 while red bars indicate metabolites that were significantly enriched in P32 skin. AA, arachidonic acid; LA, linoleic acid; DHA, docosahexaenoic acid; DHGLA, dihomo-gamma-linoleic aid; EPA, eicosapentaenoic acid; EET, epoxyeicosatrienoic acid; DHET, dihydroxyicosatrienoic acid; EpOME, epoxyoctadecenoic acid; DiHOME, dihydroxyoctadecenoic acid; DHDP, dihydroxyocosapentaenoic acid; PG, prostaglandin; TX, thromboxane; LTB4, leukotriene B4; HETE, hydroxyeicosatetraenoic acid; HDHA hydroxydocosahexaenoic acid; oxoETE, oxo-eicosatetraenoic acid; HOTrE, hydroxyoctadecatrienoic acid; HEPE, hydroxyeicosapentaenoic acid; HpODE, hydroperoxyoctadecadienoic acid; HODE, hydroxyoctadecadienoic acid.

a

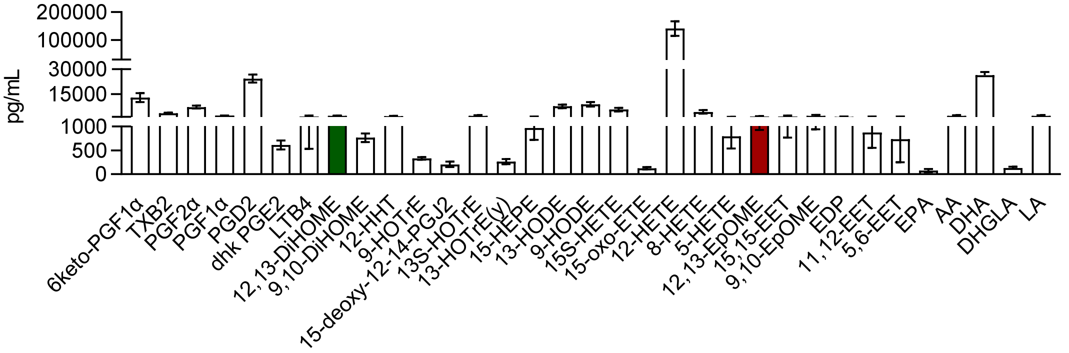

b

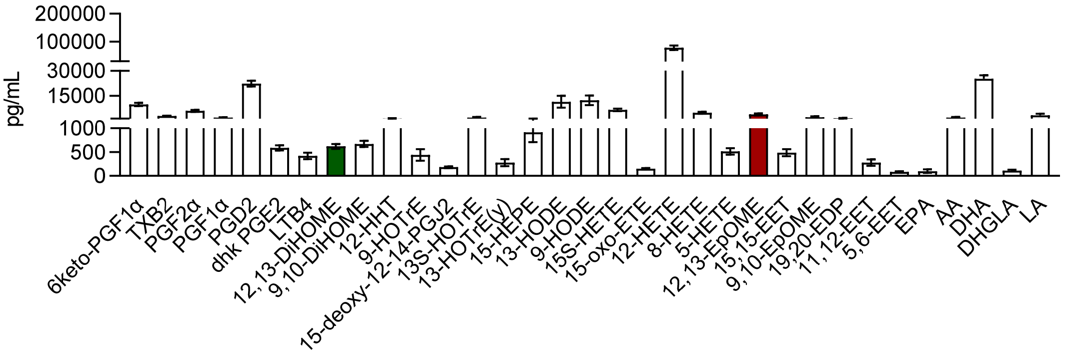

Supplementary Fig. 3. Concequences of sEH deletion on PUFA-derived mediators. PUFA-derived mediators in hair follicles isolated from (**a**) wild-type and (**b**) sEH$^{-/-}$ mice; n=4-5 mice per group with 25 follicles per mouse. n=5-8 mice per group; Green bars indicate metabolites that were significantly higher in wild-type hair follicles while red signifies the metabolites enriched in in sEH$^{-/-}$ follicles**.** AA, arachidonic acid; LA, Linoleic acid; DHA, Docosahexaenoic acid; DHGLA, dihomo-gamma-linoleic aid; EPA, Eicosapentaenoic acid; EET, epoxyeicosatrienoic acid; DHET, dihydroxyicosatrienoic acid; EpOME, epoxyoctadecenoic acid; DiHOME, dihydroxyoctadecenoic acid; DHDP, dihydroxyocosapentaenoic acid; PG, prostaglandin; TX, Thromboxane; LTB4, Leukotriene B4; HETE, hydroxyeicosatetraenoic acid; HDHA hydroxydocosahexaenoic acid; oxoETE, oxo-eicosatetraenoic acid; HOTrE, hydroxyoctadecatrienoic acid; HEPE, hydroxyeicosapentaenoic acid; HpODE, hydroperoxyoctadecadienoic acid; HODE, hydroxyoctadecadienoic acid

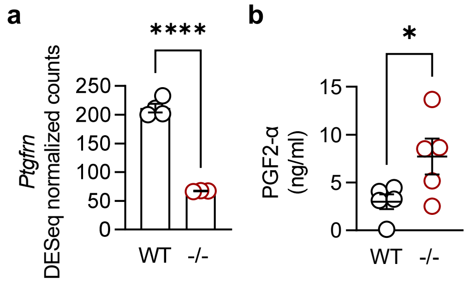

Supplementary Fig. 4. Consequence of sEH deletion on ptgfrn expression and PGF2α levels. (**a**) Ptgfrn expression (RNA-seq) in anagen staged (P32) dorsal skin from wild-type (WT) and sEH$^{-/-}$ (-/-) mice; n=3-4 mice per group (Student's t test). (**b**) PGF2α levels in anagen staged (P32) dorsal skin from wild-type (WT) and sEH$^{-/-}$ (-/-) mice; n=5 mice per group (Student's t test). *P<0.05, ****P<0.0001.
